# Bile acid signaling, metabolism, and aging

**DOI:** 10.1016/j.livres.2026.02.002

**Published:** 2026-02-10

**Authors:** Ji Sun, Shili Zhang, Lihua Jin, Wendong Huang

**Affiliations:** a Department of Diabetes Complications and Metabolism, Arthur Riggs Diabetes and Metabolism Research Institute, Beckman Research Institute, City of Hope National Medical Center, Duarte, CA, USA; b Department of Endocrinology, Zhongda Hospital, School of Medicine, Southeast University, Nanjing, Jiangsu, China; c Department of Endocrinology, Guangdong Provincial People’s Hospital (Guangdong Academy of Medical Sciences), Southern Medical University, Guangzhou, Guangdong, China

**Keywords:** Aging, Bile acids (BAs), Gut microbiota, Metabolic disorders, Neurodegeneration

## Abstract

Bile acids (BAs) serve not only as key facilitators of lipid absorption but also as crucial signaling molecules regulating glucose and lipid metabolism, inflammation, and overall energy homeostasis. Aging profoundly alters BA metabolism, characterized by shifts in biosynthetic pathways, compositional changes, disrupted receptor-mediated signaling, and alterations in gut microbiota interactions. These age-related changes contribute to the onset and progression of metabolic conditions, including type 2 diabetes, obesity, metabolic dysfunction-associated fatty liver disease, and neurodegenerative disorders. An increased abundance of hydrophobic and cytotoxic BAs has been associated with systemic inflammation, metabolic rigidity (disruption of metabolic flexibility), and organ dysfunction. Targeting BA signaling—through pharmacological modulation of farnesoid X receptor and Takeda G protein-coupled receptor 5 or microbiota-directed therapies—offers promising strategies to mitigate aging-related metabolic decline. A deeper understanding of how BA metabolism evolves over the lifespan may unveil novel interventions to promote healthy aging and prevent age-related disease.

## Highlights

• Aging increases 12α-hydroxylated bile acids, raising hydrophobicity and metabolic risk.
• FXR and TGR5 signaling decline with age, impairing metabolic homeostasis.
• Gut dysbiosis remodels bile acids, driving barrier dysfunction and inflammation.
• Targeting bile acid pathways may mitigate aging-related metabolic and neurodegenerative disorders.

## Graphical abstract

**Figure:**
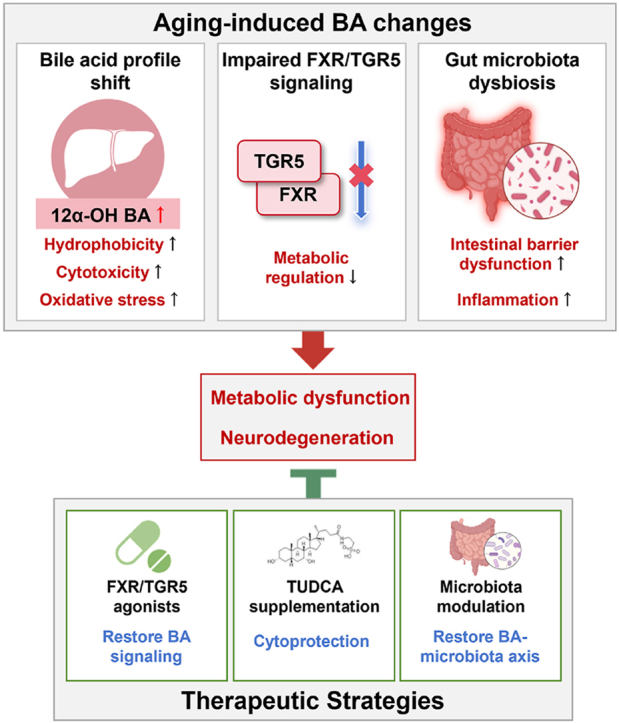

## 1 Introduction

Aging is characterized by a gradual decline in metabolic homeostasis, manifested as insulin resistance, dyslipidemia, chronic low-grade inflammation (inflammaging), and increased susceptibility to degenerative diseases. In the United States, 90% of individuals aged 65 and older suffer from age-associated chronic conditions, almost 80% of older adults have impaired glycemic control, and over 20% are affected by type 2 diabetes (T2D).^1^^,^^2^ A similarly high metabolic disease burden is observed in China, where more than 60% of adults aged 60 years and older are prediabetic or diabetic, and over half are affected by dyslipidemia.^3^^,^^4^ There is still an unmet need to identify the root causes of age-related metabolic dysfunction and to develop safer and more effective therapies that halt and prevent disease progression.

Bile acids (BAs) are amphipathic molecules synthesized from cholesterol in the liver. Beyond their classical role in lipid emulsification and absorption, BAs function as signaling molecules that regulate diverse physiological processes, including glucose and lipid metabolism, energy balance, immune responses, and intestinal barrier integrity.^5^ These regulatory effects are mediated primarily through interactions with BA receptors, such as the farnesoid X receptor (FXR) and Takeda G protein-coupled receptor 5 (TGR5), which exert context-dependent effects on downstream signaling pathways.^6^ Emerging evidence suggests that BA metabolism undergoes significant remodeling during aging, including alterations in its synthesis pathway, composition, receptor-mediated signaling, and gut microbiota interactions.^7^ Dysregulation of these processes contributes to the disruption of metabolic balance, exacerbating the development of aging-related diseases such as T2D, metabolic dysfunction-associated fatty liver disease (MAFLD), and neurodegeneration. However, the role of BAs in age-associated conditions remains unclear.

This review provides a comprehensive overview of age-associated alterations in BA composition, microbiota-mediated BA transformation, and receptor-dependent signaling. We also discuss the implications of these changes for the pathophysiology of metabolic and neurodegenerative diseases and highlight therapeutic strategies targeting BA pathways to promote healthy aging.

## 2 Age-related changes in BA metabolism

**Fig. 1:**
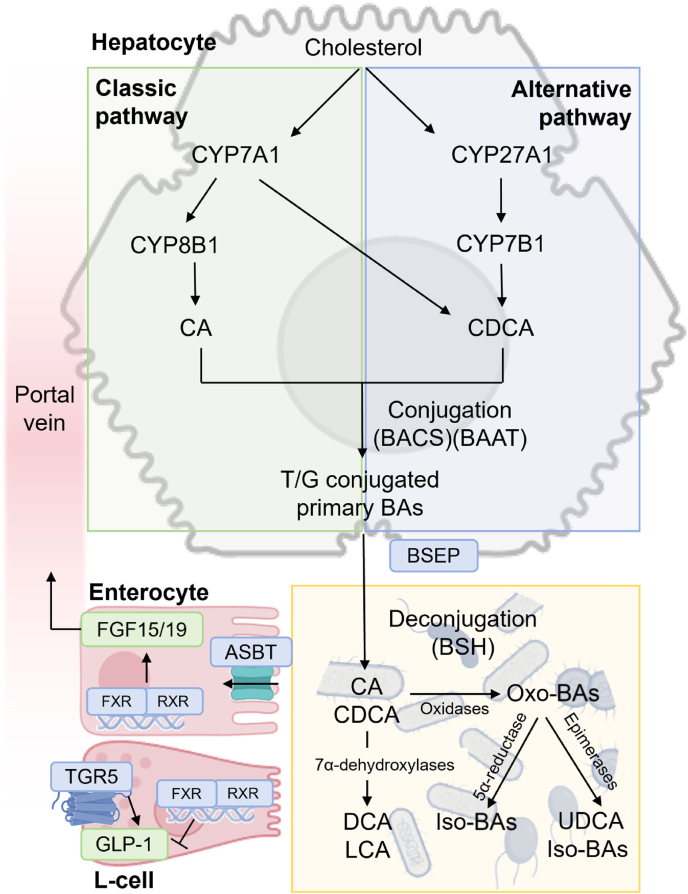
**Overview of BA synthesis, gut microbiome-mediated transformation, and enterohepatic circulation.** Primary BAs, including CA and CDCA, are synthesized from cholesterol in hepatocytes via two main pathways: the classic pathway, initiated by CYP7A1, which generates 12α-hydroxylated BAs such as CA with CYP8B1 activity, and non-12α-hydroxylated BAs such as CDCA in the absence of CYP8B1 activity; and the alternative pathway, initiated by CYP27A1, which produces non-12α-hydroxylated BAs such as CDCA. These BAs are then conjugated with glycine or taurine via BACS and BAAT to form bile salts and secreted into the bile canaliculi through the BSEP. Upon release into the intestine after a meal, conjugated BAs undergo microbiome-mediated biotransformation. This includes deconjugation by BSH, 7α-dehydroxylation by 7α-dehydroxylases to generate secondary BAs such as DCA and LCA, followed by further modifications, including oxidation-reduction, epimerization, and 5α-reduction, catalyzed by oxidases, epimerases, and 5α-reductase, respectively. These reactions contribute to the formation of UDCA and iso-BAs. In the terminal ileum, BAs are actively reabsorbed by enterocytes via the ASBT, where FXR activation induces FGF15/19, which signals to the liver to suppress CYP7A1-and CYP8B1-mediated BA synthesis. In enteroendocrine L-cells, TGR5 activation by secondary BAs promotes GLP-1 secretion. The recirculated BAs return to hepatocytes via the portal vein, completing the enterohepatic circulation. Abbreviations: ASBT, apical sodium-dependent bile acid transporter; BAAT, bile acid-CoA: amino acid N-acyltransferase; BACS, bile acid-CoA synthetase; BAs, bile acids; BSH, bile salt hydrolase; BSEP, bile salt export pump; CA, cholic acid; CDCA, chenodeoxycholic acid; CYP7A1, cholesterol 7α-hydroxylase; CYP8B1, sterol 12α-hydroxylase; CYP27A1, sterol 27-hydroxylase; DCA, deoxycholic acid; FGF15/19, fibroblast growth factor 15/19; FXR, farnesoid X receptor; GLP-1, glucagon-like peptide-1; LCA, lithocholic acid; RXR, retinoid X receptor; TGR5, Takeda G protein-coupled receptor 5; UDCA, ursodeoxycholic acid.

BAs are synthesized from cholesterol via a series of enzymatic reactions primarily occurring in hepatocytes. The biosynthesis, conjugation, transportation, and enterohepatic circulation of BAs are tightly regulated processes essential for maintaining lipid homeostasis and systemic metabolic balance (Fig. 1). Aging influences multiple steps of BA metabolism, resulting in compositional and functional alterations that may lead to metabolic dysregulation.

### 2.1 BA synthesis pathway

BA synthesis occurs through two major pathways: the classical pathway and the alternative pathway. The classical pathway is initiated by cholesterol 7α-hydroxylase (CYP7A1), with a key branching step catalyzed by sterol 12α-hydroxylase (CYP8B1), which determines the production of 12α-hydroxylated BAs. Specifically, CYP8B1 converts 7α-hydroxy-4-cholesten-3-one (7α-HCO or C4) into 7α,12α-dihydroxy-4-cholesten-3-one (7α,12α-DCO), a critical precursor of cholic acid (CA), a primary 12α-hydroxylated BA. In contrast, when CYP8B1 activity is limited, 7α-HCO is ultimately converted into chenodeoxycholic acid (CDCA), a primary non-12α-hydroxylated BA.^8^^,^^9^ The alternative pathway, initiated by sterol 27-hydroxylase (CYP27A1), also leads to CDCA formation but bypasses CYP8B1, resulting exclusively in non-12α-hydroxylated BAs.^10^ Thus, CYP8B1 plays a pivotal role in regulating the ratio of 12α-hydroxylated to non-12α-hydroxylated BAs, which is critical for determining their physicochemical and metabolic properties. BAs are classified as either hydrophobic or hydrophilic based on their aqueous solubility, detergent properties, and biological activity.^11^ A higher proportion of 12α-hydroxylated BA, such as CA and deoxycholic acid (DCA), enhances hydrophobicity, promoting lipid absorption and modulating the activity of multiple BA receptors, including FXR, TGR5, vitamin D receptor (VDR), and sphingosine-1-phosphate receptor 2 (S1PR2).^12^ CA and DCA are relatively weak agonists of FXR but potent activators of TGR5, particularly promoting glucagon-like peptide-1 (GLP-1) secretion and energy expenditure. In contrast, non-12α-hydroxylated BAs such as ursodeoxycholic acid (UDCA) and its glycine-conjugated form are more hydrophilic. UDCA is generally considered an FXR antagonist or weak modulator and can exert cytoprotective and anti-inflammatory effects, partly through TGR5 activation.^13^ Among naturally occurring BAs, CDCA is the most potent endogenous FXR agonist.^14^ Maintaining the balance between these BA species is essential for BA function and systemic metabolic homeostasis. Alterations in BA composition, such as those occurring with aging, can disrupt receptor signaling and contribute to systemic metabolic dysregulation.^15^

### 2.2 Conjugation and enterohepatic circulation

Following hepatic synthesis in humans, primary BAs—CA and CDCA—are conjugated with glycine or taurine by BA-CoA synthetase (BACS) and BA-CoA: amino acid N-acyltransferase (BAAT), forming bile salts such as taurocholic acid (TCA), glycocholic acid (GCA), taurochenodeoxycholic acid (TCDCA), and glycochenodeoxycholic acid (GCDCA).^16^ Conjugation enhances BA solubility and reduces passive diffusion across cellular membranes. Conjugated BAs, along with a small fraction of unconjugated BAs, are secreted into bile canaliculi via the bile salt export pump (BSEP), stored in the gallbladder, and released into the duodenum after food intake to facilitate lipid emulsification and absorption. Approximately 95% of intestinal BAs are reabsorbed in the distal ileum through the apical sodium-dependent BA transporter (ASBT) and returned to the liver via the portal vein, forming the enterohepatic circulation, which recycles BAs multiple times daily.^17^ A small portion escapes reabsorption and enters the distal ileum and colon, where gut microbiota convert them into secondary BAs—such as DCA and lithocholic acid (LCA)—via deconjugation and 7α-dehydroxylation.^18^ Additional modifications, including epimerization and oxidation–reduction reactions, generate BA derivatives such as UDCA, the 7β-epimer of CDCA. These secondary and modified BAs may be passively reabsorbed or excreted in feces, contributing to the compositional diversity and dynamic regulation of the BA pool.

The integrity of enterohepatic circulation is essential for BA homeostasis. With aging, impaired conjugation, transportation, or recycling efficiency disrupts lipid metabolism, weakens gut barrier function, and promotes systemic inflammation, thereby contributing to metabolic and age-related diseases.^13^^,^^19^

### 2.3 Impact of aging on BA metabolism

Aging profoundly alters BA composition, impacting both BA synthesis and enterohepatic circulation, and thereby contributing to the metabolic dysfunction and chronic inflammation commonly observed in the elderly. In the liver, aging is associated with a decline in the expression and activity of key enzymes involved in BA biosynthesis. Notably, expression of CYP7A1, the rate-limiting enzyme of the classical pathway, declines significantly with age, resulting in decreased overall BA production.^20^ Specifically, aging is associated with a shift toward a more 12α-hydroxylated bile acid profile. In aged rats, increased CA and decreased β-MCA levels suggest reduced conversion of CDCA into β-MCA and a simultaneous shift toward 12α-hydroxylated BA synthesis, resulting in an elevated CA/CDCA ratio.^21^ A clinical cross-sectional study revealed that BA profiles, particularly CDCA and its conjugates, are significantly influenced by both gender and age.^19^ Alterations in BA composition modulate the activity of BA receptors, thereby influencing metabolic regulation and inflammatory responses.

Aging also alters BA conjugation patterns, affecting the relative proportions of taurine- and glycine-conjugated species.^22^ Beyond changes in conjugation, aging induces a shift toward a more hydrophobic and cytotoxic BA pool. Elderly individuals display altered compositions of both primary and secondary BAs, characterized by elevated levels of secondary species such as DCA and taurolithocholic acid (TLCA).^23^^,^^24^ The accumulation of these hydrophobic BAs enhances membrane-disruptive properties, promotes oxidative stress, and exacerbates inflammatory responses, ultimately impairing metabolic regulation and tissue homeostasis.^11^^,^^25^ Moreover, aging disrupts the enterohepatic circulation of BAs. Ren *et al*.^26^ observed elevated serum levels of conjugated primary BAs in both elderly individuals and rodents, which were associated with increased expression of the ileal ASBT. Collectively, these age-induced alterations in BA synthesis, composition, and circulation suggest a critical role of BAs in aging and age-related disorders.

## 3 Gut microbiota-BA interactions in aging

The gut microbiota critically shapes BA composition and function through microbial transformations. Aging-associated dysbiosis disrupts this process, leading to BA imbalance and metabolic decline. Conversely, BAs influence microbial composition via antimicrobial and immunomodulatory effects. This bidirectional interaction between BAs and the microbiota plays a pivotal role in modulating aging trajectories.

### 3.1 Microbiome-mediated modifications of BAs

Beyond hepatic synthesis, BA composition is significantly shaped by gut microbiota-mediated transformations. Upon entering the intestine, conjugated primary BAs undergo microbial enzymatic reactions, including deconjugation, dehydroxylation, oxidation, and epimerization, resulting in the generation of secondary BAs such as DCA, LCA, and various oxo-BAs.^27^^,^^28^

Bile salt hydrolases (BSHs), broadly expressed by commensal bacterial genera such as *Lactobacillus*, *Bifidobacterium*, *Clostridium*, and *Bacteroides*, catalyze the deconjugation of glycine- and taurine-conjugated BAs.^29^^,^^30^ Following deconjugation, specific anaerobic bacteria, such as *Clostridium scindens*, mediate dehydroxylation at the 7α-position of BA derivatives, leading to the production of hydrophobic secondary BAs with distinct signaling and cytotoxic properties.^31^^,^^32^ Recent studies have expanded our understanding of microbiota-mediated BA metabolism. Certain gut microbes not only deconjugate but also reconjugate BAs with amino acids other than glycine and taurine, forming a novel class of BAs known as microbially conjugated BAs (MCBAs). These conjugates include phenylalanocholic acid, tyrosocholic acid, and leucocholic acid, as well as conjugates with citrulline, glutamic acid, histidine, isoleucine, leucine, lysine, threonine, tryptophan, and tyrosine, which were identified in human and murine intestinal samples.^33^^,^^34^ The reconjugation process is primarily mediated by certain BSHs expressed by gut bacteria such as *Lactobacillaceae, Bacteroides fragilis,* and *Eggerthella lenta*. These microbial modifications significantly alter the binding affinity and agonist/antagonist activity of BAs toward receptors such as FXR and TGR5. For instance, phenylalanocholic acid and tyrosocholic acid exhibit much stronger FXR agonist activity than CDCA *in vitro*, highlighting the immunomodulatory potential of MCBAs in reshaping host metabolism and immune responses.^32^^,^^35^

### 3.2 Aging-associated microbial dysbiosis and BA alterations

Aging is associated with profound alterations in gut microbiota composition, commonly referred to as microbial dysbiosis. Characteristic features of age-associated dysbiosis include a decline in the abundance of some beneficial BA-transforming bacteria, such as *Clostridium* *spp.* and *Bifidobacterium* *spp.*, and an expansion of opportunistic or pro-inflammatory taxa.^36^, ^37^, ^38^ The reduction of key BA-modifying bacteria impairs secondary BA production, which may contribute to a shift in BA composition toward increased hydrophobicity and proinflammatory potential.^39^ This imbalance compromises intestinal barrier integrity, promotes endotoxemia, and triggers systemic low-grade inflammation.^40^ Notably, specific bacterial species enriched in centenarians, such as *Alistipes putredinis* and *Odoribacter splanchnicus*, produce unique secondary BAs like isoallo-LCA, which have been implicated in maintaining gut barrier function and resisting pathogen colonization.^7^^,^^41^ These findings underscore the pivotal role of microbiota-driven BA metabolism in modulating aging trajectories and health span.

### 3.3 BAs as modulators of gut microbiota and aging physiology

Conversely, BAs actively shape gut microbial composition and function, influencing aging-related intestinal and systemic physiology. Hydrophobic BAs, particularly secondary BAs like DCA and LCA, exert potent antimicrobial activity by disrupting bacterial membranes, thereby selectively suppressing or enriching microbial taxa based on their BSH activity and bile resistance.^42^ For instance, high concentrations of DCA have been shown to inhibit BSH-producing genera such as *Lactobacillus* and *Clostridium*.^43^ Conversely, certain BA profiles can support the growth of beneficial microbes such as *Akkermansia muciniphila* and *Bifidobacterium* species, known for enhancing gut barrier integrity and exerting anti-inflammatory effects.^44^^,^^45^ Moreover, secondary BAs and their derivatives (*e.g.,* 3-oxoLCA, isoalloLCA) serve as signaling molecules that modulate host immunity by influencing T cell differentiation and cytokine production.^46^ These reciprocal interactions between BAs and gut microbiota establish a dynamic feedback loop that can either exacerbate or ameliorate aging-associated intestinal inflammation and systemic dysfunction, depending on the composition, transformation, and signaling context of the BA pool.

## 4 BA receptor signaling in metabolic regulation

The effects of BAs are primarily mediated through two key receptors, FXR and TGR5. Proper regulation of these receptor pathways is essential for maintaining systemic metabolic balance, and disruptions in their signaling are increasingly linked to age-associated metabolic dysfunction.

### 4.1 FXR: a nuclear BA receptor governing BA and metabolic homeostasis

FXR is predominantly expressed in the liver and intestine, where it plays a central role in maintaining BA homeostasis by regulating their synthesis, transport, and detoxification.^47^^,^^48^ Upon activation by BAs—particularly CDCA—intestinal FXR induces the expression of fibroblast growth factor-15/19 (FGF15 in mice; FGF19 in humans), which is secreted into the portal circulation and binds to the FGFR4/β-Klotho receptor complex on hepatocytes.^49^ This endocrine signaling suppresses the expression of CYP7A1 and CYP8B1, the key enzymes in the classical BA synthesis pathway.^50^ In parallel, hepatic FXR directly induces the expression of small heterodimer partner (SHP), a transcriptional corepressor that interacts with liver receptor homolog-1 (LRH-1) and hepatocyte nuclear factor 4α (HNF4α) to further inhibit CYP7A1 transcription.^51^ FXR also transcriptionally regulates several BA transporters, including BSEP, the organic solute transporter α/β (OSTα/β), and the Na^+^/taurocholate cotransporting polypeptide (NTCP) in the liver, as well as ASBT and ileal BA-binding protein (IBABP) in the ileum, thus coordinating BA circulation and enterohepatic recycling.^52^^,^^53^ Beyond its role in BA metabolism, the FXR–FGF15/19 axis influences systemic energy homeostasis by inhibiting hepatic gluconeogenesis, stimulating glycogen and protein synthesis via extracellular signal-regulated protein kinase 1/2 (ERK1/2) signaling, and promoting gallbladder filling.^54^^,^^55^ FXR activation also improves lipid profiles, enhances insulin sensitivity, and exerts anti-inflammatory effects through suppression of nuclear factor kappa B (NF-κB)-mediated gene transcription.^56^^,^^57^

During aging, FXR signaling responsiveness appears to be attenuated, contributing to the metabolic dysregulation and chronic low-grade inflammation that characterize the aging process.^58^ Therapeutic targeting of FXR has thus been proposed as a potential strategy to promote healthy aging and longevity.^59^ Obeticholic acid (OCA), a semi-synthetic agonist of FXR, exerts multifaceted benefits by targeting several age-related metabolic and inflammatory dysfunctions. It suppresses hepatic lipogenesis, alleviates inflammation, improves liver function, and reduces hepatic fibrosis.^60^ Additionally, OCA enhances reverse cholesterol transport and insulin sensitivity, contributing to systemic metabolic improvement.^61^ Moreover, an intestine-specific FXR agonist, fexaramine, has been shown to increase skeletal muscle mass and improve muscle performance in aged mice, offering metabolic benefits while minimizing systemic side effects.^58^^,^^62^ Thus, preserving or restoring FXR signaling function may represent a potential therapeutic approach to mitigate age-associated metabolic decline and promote healthy longevity.

### 4.2 TGR5: a membrane BA receptor modulating energy homeostasis and inflammation

TGR5 is a membrane-bound G protein-coupled BA receptor widely expressed in metabolic and immune-related tissues, including the intestine, adipose tissue, liver, skeletal muscle, and the central nervous system.^63^ TGR5 is preferentially activated by secondary BAs such as LCA and DCA.^14^^,^^64^ Activation of TGR5 promotes cyclic adenosine monophosphate (cAMP) production, subsequently activating protein kinase A (PKA) and downstream transcription factors such as cAMP-response element-binding protein (CREB).^65^ These signaling events enhance thermogenesis and energy expenditure, particularly through upregulation of uncoupling protein-1 (UCP1) in brown adipose tissue (BAT).^66^ In the intestine, TGR5-mediated signaling stimulates GLP-1 secretion from enteroendocrine L-cells, facilitating improved insulin sensitivity and glucose metabolism.^67^ Furthermore, TGR5 activation modulates immune cell function, reducing the production of proinflammatory cytokines.^68^

In the context of aging, studies suggest that TGR5-mediated signaling becomes impaired, leading to diminished energy homeostasis and reduced anti-inflammatory capacity.^69^^,^^70^ Pharmacological activation of TGR5 has thus emerged as a promising strategy to combat age-associated metabolic dysfunction. TGR5 agonists, such as INT-777 and BAR501, have been shown to enhance energy expenditure, stimulate GLP-1 secretion, and attenuate inflammatory responses.^67^^,^^71^ Together, these findings underscore the therapeutic potential of TGR5 activation in restoring metabolic balance and immune resilience during aging.

### 4.3 Temporal and tissue-specific interactions between FXR and TGR5

Although BAs activate both FXR and TGR5, they mediate distinct yet complementary physiological effects depending on tissue context and timing. FXR and TGR5 are coexpressed in enteroendocrine L-cells, where FXR activation has been shown to upregulate TGR5 transcription through a direct binding site in the TGR5 promoter region.^72^ In the intestine, TGR5 activation rapidly promotes GLP-1 secretion postprandially, while FXR activation induces a delayed transcriptional repression of GLP-1 gene expression.^73^^,^^74^ This sequential activation ensures the fine-tuning of glucose metabolism during nutrient intake. In metabolic tissues such as the liver and adipose tissue, coordinated FXR and TGR5 signaling supports lipid clearance, thermogenesis, and inflammation control.^72^^,^^75^ However, aging disrupts this balance, contributing to metabolic inflexibility, chronic low-grade inflammation, and increased vulnerability to aging-related metabolic and neurodegenerative diseases.^69^ To counteract these impairments, dual FXR/TGR5 agonists such as INT-767 have been developed, demonstrating synergistic benefits in improving metabolic parameters, reducing hepatic steatosis, and alleviating systemic inflammation in preclinical models.^76^ Nonetheless, translating these findings into clinical therapies requires careful evaluation of long-term efficacy, tissue-specific targeting, and safety, particularly in elderly populations with complex comorbidities. A deeper understanding of the spatiotemporal coordination between FXR and TGR5 is essential for elucidating the role of BA signaling in systemic metabolism and for developing targeted interventions to combat aging-related pathologies.

## 5 Role of BAs in age-associated metabolic disorders

**Fig. 2:**
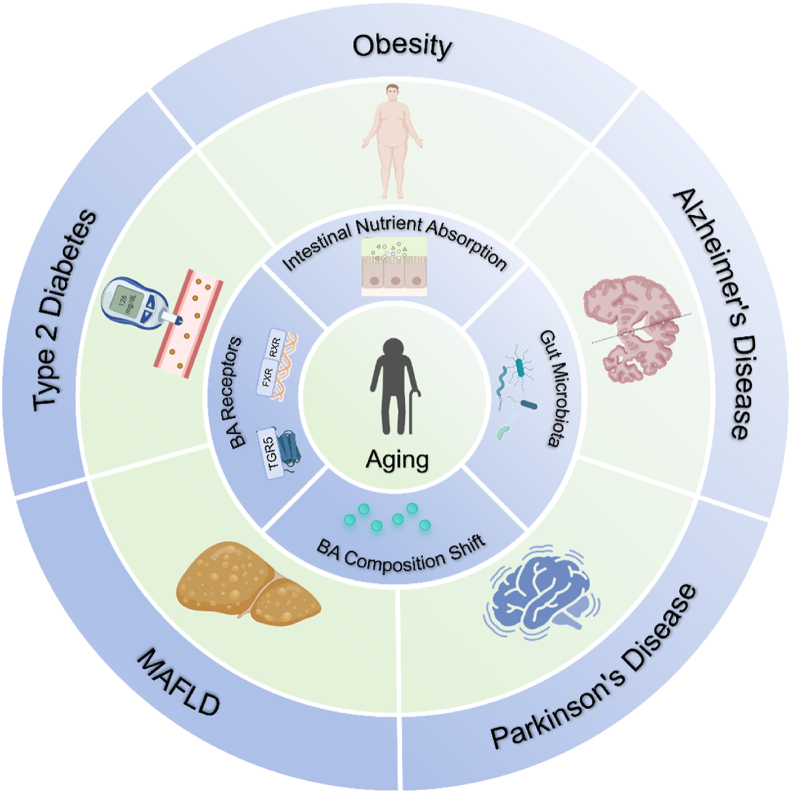
**Age-associated BA dysregulation links to multiple chronic diseases.** Aging induces significant shifts in gut microbiota composition, leading to microbial dysbiosis and subsequent disruption of BA metabolism. These changes include altered BA composition, which in turn affects signaling through key BA receptors, including FXR and TGR5. Reduced receptor activation disrupts metabolic regulation, energy homeostasis, immune balance, and neuroprotection. These molecular alterations contribute to the development and progression of multiple aging-related diseases, including T2D, obesity, MAFLD, AD, and PD. Therapeutic interventions such as BA supplementation and receptor-specific agonists may help restore BA homeostasis and mitigate age-associated metabolic and neurodegenerative pathologies. Abbreviations: AD, Alzheimer’s disease; BA, bile acid; FXR, farnesoid X receptor; MAFLD, metabolic dysfunction-associated fatty liver disease; PD, Parkinson’s disease; RXR, retinoid X receptor; T2D, type 2 diabetes; TGR5, Takeda G protein-coupled receptor 5.

Alterations in BA composition and receptor-mediated signaling during aging contribute to the development and progression of multiple metabolic disorders. Dysregulated BA metabolism affects not only liver and intestinal physiology, but also impacts systemic glucose homeostasis, lipid metabolism, and neural integrity. This chapter discusses the role of BAs in aging-associated diseases, including T2D, obesity, MAFLD, Alzheimer’s disease (AD), and Parkinson’s disease (PD), highlighting the mechanistic links between BA dysregulation and metabolic pathologies (Fig. 2).

### 5.1 T2D and insulin resistance

The prevalence of T2D increases markedly with age, largely driven by cellular senescence, β-cell dysfunction, and insulin resistance.^77^ BAs regulate glucose metabolism via FXR and TGR5, which modulate hepatic gluconeogenesis and incretin hormone secretion, respectively. Specifically, FXR regulates hepatic gluconeogenesis and insulin sensitivity, while TGR5 stimulates GLP-1 secretion from enteroendocrine cells, enhancing postprandial insulin release.^78^^,^^79^ However, aging disrupts this regulation. Reduced FXR expression and diminished TGR5 activity in elderly individuals impair insulin signaling pathways.^80^^,^^81^ Furthermore, changes in BA composition, including increased levels of certain hydrophobic and potentially cytotoxic species, have been linked to heightened systemic inflammation and oxidative stress, which may impair β-cell function and insulin signaling in peripheral tissues.^82^^,^^83^ These mechanisms collectively accelerate the onset and progression of T2D in aging individuals.

Recent preclinical studies underscore the therapeutic potential of BA modulation. FXR agonists such as GW4064 and the gut-restricted fexaramine improve glucose metabolism in preclinical models.^74^^,^^78^ Tauroursodeoxycholic acid (TUDCA), a hydrophilic BA, enhances insulin secretion via TGR5-cAMP-PKA signaling and significantly improves glucose tolerance and insulin clearance in aged mice by enhancing hepatic insulin-degrading enzyme expression, reducing adiposity, and promoting energy expenditure.^84^^,^^85^ These findings underscore the potential of BA-based interventions to mitigate aging-induced insulin resistance.

### 5.2 Obesity and dyslipidemia

BAs are key regulators of systemic energy homeostasis through their modulation of lipid absorption, hepatic lipid metabolism, and adipose tissue thermogenesis. Activation of TGR5 in brown adipose tissue stimulates thermogenic gene expression and enhances energy expenditure via a cAMP-dependent pathway, involving increased mitochondrial activity and thyroid hormone activation.^86^^,^^87^ Concurrently, FXR activation limits the expansion of white adipose tissue and promotes lipid metabolism by suppressing hepatic lipogenesis and enhancing fatty acid oxidation and triglyceride clearance.^88^^,^^89^ Aging disrupts BA signaling by decreasing FXR/TGR5 expression and shifting BA composition toward hydrophobic species like CA and DCA.^90^ These changes promote lipid accumulation, visceral adiposity, and proinflammatory adipocyte phenotypes. Moreover, elevated CA/DCA levels may trigger hepatic lipid peroxidation and inflammation, aggravating metabolic inflexibility.^91^^,^^92^

BA-based interventions confer metabolic benefits. UDCA has been reported to alleviate age-related metabolic disturbances by decreasing hepatic lipid buildup and reducing the inflammatory responsiveness of hepatocytes and adipocytes.^93^ Additionally, FXR activation in aged mice regulates lipid metabolism and maintains energy homeostasis, thereby contributing to lifespan extension.^59^ Modulating BA transporters—particularly those affected by aging such as ASBT and NTCP—or microbial enzymes involved in BA deconjugation may influence BA absorption and systemic availability, thereby impacting age-associated metabolic outcomes, including obesity and dyslipidemia.^94^ These insights highlight the pivotal role of BA signaling pathways in maintaining metabolic flexibility and suggest that targeting FXR and TGR5 may offer therapeutic opportunities to combat obesity and dyslipidemia during aging.

### 5.3 MAFLD

MAFLD is a growing global health issue linked to obesity, metabolic syndrome, and insulin resistance. Disruptions in BA homeostasis are common in MAFLD, and BA profiles are emerging as biomarkers of disease severity. Accumulating evidence underscores the pivotal role of BAs in MAFLD development and progression through their interactions with nuclear receptors like FXR and TGR5.^95^ In MAFLD patients, increased levels of primary 12α-hydroxylated (OH) BAs—such as CA and DCA—are driven by upregulated CYP8B1, leading to a higher 12-OH/non-12-OH BA ratio. Concomitantly, protective non-12-OH BAs such as UDCA and LCA are reduced, promoting hepatic lipid accumulation, steatosis, and inflammation.^96^^,^^97^

Aging is increasingly recognized as a key risk factor in MAFLD development. The concentration of the secondary BA DCA increases with age and is significantly elevated in elderly individuals and aged male mice compared to their younger counterparts.^15^^,^^98^ Excessive DCA generates reactive oxygen species that cause DNA damage and promote a senescence-associated secretory phenotype, thereby accelerating disease progression.^99^ Moreover, FXR plays a complex role in age-related hepatic steatosis. While aged FXR-deficient mice exhibit increased energy expenditure and better glucose regulation, they also develop severe metabolic dysfunction-associated steatohepatitis-like liver damage.^100^ Hepatic deletion of the FXR/SHP axis in older mice improves metabolic parameters and counteracts age-associated weight gain and insulin resistance.^101^ Concurrently, age-associated gut microbiota dysbiosis increases the ratio of unconjugated to conjugated BAs, elevating BA hydrophobicity and cytotoxicity.^102^ This shift enhances the generation of reactive oxygen species, DNA damage, apoptosis, and necrosis.^103^ Dysregulation of the gut-liver axis further promotes intestinal barrier dysfunction, systemic inflammation, and hepatic injury.^104^^,^^105^ These combined effects are implicated in the pathogenesis of age-related disorders, particularly those involving dysregulated lipid metabolism.

Targeting BA signaling represents a promising therapeutic avenue for aging-related MAFLD. Pharmacological activation of FXR (*e.g.,* OCA) and microbiota-directed strategies to restore a favorable BA profile are actively under investigation.^106^^,^^107^ In preclinical models, administration of BAR502, a dual FXR/TGR5 agonist, was shown to promote browning of white adipose tissue and reduce hepatic steatosis and fibrosis.^108^ Collectively, these findings emphasize the therapeutic potential of modulating BA signaling to counteract MAFLD progression in aging populations.

### 5.4 Neurodegenerative diseases

Emerging evidence has identified BA metabolism as a critical factor implicated in the pathogenesis of neurodegenerative diseases, including AD and PD.^109^^,^^110^ BAs, synthesized primarily in the liver, can enter the systemic circulation and cross the blood-brain barrier via passive diffusion or transporter-mediated mechanisms, directly interacting with neurons and glial cells.^111^ Within the central nervous system, BAs exert their effects through activation of FXR and TGR5, which are expressed in neurons, astrocytes, and microglia.^79^^,^^112^^,^^113^ These receptors play vital roles in regulating inflammation, energy metabolism, and neuronal survival.

During aging, perturbations in BA homeostasis, particularly the accumulation of hydrophobic and cytotoxic secondary BAs such as DCA and LCA, promote neuroinflammation, oxidative stress, and mitochondrial dysfunction, all of which are hallmark features of neurodegenerative processes.^114^, ^115^, ^116^ In AD patients, elevated levels of bacterially derived DCA and its conjugated forms (glycine and taurine) have been observed in serum, while increased intestinal levels of DCA and LCA have been reported in PD patients.^117^^,^^118^ Impaired FXR and TGR5 signaling further exacerbate neuronal vulnerability by disrupting energy metabolism, impairing synaptic plasticity, and dysregulating inflammatory responses.^119^^,^^120^

Certain BAs show neuroprotective potential. UDCA and its conjugate, glycoursodeoxycholic acid (GUDCA), protect brain endothelial cells by reducing apoptotic cell death.^121^ Moreover, microglial expression of TGR5 enables anti-inflammatory effects upon binding with TUDCA, as demonstrated in mouse models of acute brain inflammation.^122^ TGR5 activation by agonists such as INT-777 has also been shown to improve memory deficits and attenuate inflammation and apoptosis in AD models.^123^ Additionally, peripheral TGR5-mediated GLP-1 release, capable of crossing the blood-brain barrier, may indirectly support brain health.^124^ Low FXR expression appears to mitigate Aβ-induced neuronal apoptosis and prevent the downregulation of CREB and brain-derived neurotrophic factor.^125^ Therefore, targeting BA signaling pathways through pharmacological modulation of FXR and TGR5 activities or supplementation with cytoprotective BAs such as TUDCA may represent potential therapeutic strategies for mitigating neurodegeneration and preserving cognitive function in aging populations.

## 6 Conclusions and future perspectives

Aging is associated with profound alterations in BA metabolism, encompassing changes in BA synthesis pathways, BA levels, and composition. These changes may impair intestinal nutrient absorption, disrupt BA receptor-mediated signaling, and alter gut microbiota composition and functions. These multidimensional age-related disruptions in BA could contribute significantly to the onset and progression of metabolic, inflammatory, and neurodegenerative diseases as commonly observed in elderly populations.

Emerging evidence highlights the therapeutic potential of modulating BA metabolism to counteract age-associated disorders. Strategies under investigation include pharmacological activation of FXR and TGR5, dual agonist approaches, supplementation with cytoprotective BAs such as TUDCA, and gut microbiota-based interventions to restore BA homeostasis and BA receptor signaling.

Future research should focus on longitudinal studies to clarify the temporal dynamics of BA dysregulation throughout the lifespan, explore the crosstalk between BA signaling and other metabolic or immune pathways, and develop personalized interventions based on individual BA signatures. These efforts may pave the way for innovative therapies that promote healthy aging, prevent chronic disease, and extend health span.

## Authors’ contributions

**Ji Sun:** Writing – original draft, Resources, Conceptualization. **Shili Zhang:** Writing – review & editing, Writing – original draft, Resources. **Lihua Jin:** Writing – review & editing, Writing – original draft, Resources, Conceptualization. **Wendong Huang:** Writing – review & editing, Supervision, Funding acquisition, Conceptualization.

## Declaration of competing interest

The authors declare no conflicts of interest.
